# Validation of overnight oximetry to diagnose patients with moderate to severe obstructive sleep apnea

**DOI:** 10.1186/s12890-015-0017-z

**Published:** 2015-03-20

**Authors:** Liang-Wen Hang, Hsiang-Ling Wang, Jen-Ho Chen, Jiin-Chyr Hsu, Hsuan-Hung Lin, Wei-Sheng Chung, Yung-Fu Chen

**Affiliations:** Sleep Medicine Center, Department of Internal Medicine, China Medical University Hospital, Taichung, Taiwan; Department of Respiratory Therapy, College of Health Care, China Medical University, Taichung, Taiwan; Department of Beauty Science, National Taichung University of Science and Technology, Taichung, Taiwan; Department of Health Services Administration, China Medical University, Taichung, Taiwan; Department of Internal Medicine, Taipei Hospital, Ministry of Health and Welfare, New Taipei City, Taiwan; Department of Management Information System, Central Taiwan University of Science and Technology, Taichung, Taiwan; Department of Internal Medicine, Taichung Hospital, Ministry of Health and Welfare, Taichung, Taiwan; Department of Healthcare Administration, Central Taiwan University of Science and Technology, Taichung, Taiwan; Department of Dental Technology and Materials Science, Central Taiwan University of Science and Technology, Taichung, Taiwan

**Keywords:** Obstructive sleep apnea (OSA), Oximetry, Support vector machine (SVM), Polysomnography (PSG)

## Abstract

**Background:**

Polysomnography (PSG) is treated as the gold standard for diagnosing obstructive sleep apnea (OSA). However, it is labor-intensive, time-consuming, and expensive. This study evaluates validity of overnight pulse oximetry as a diagnostic tool for moderate to severe OSA patients.

**Methods:**

A total of 699 patients with possible OSA were recruited for overnight oximetry and PSG examination at the Sleep Center of a University Hospital from Jan. 2004 to Dec. 2005. By excluding 23 patients with poor oximetry recording, poor EEG signals, or respiratory artifacts resulting in a total recording time less than 3 hours; 12 patients with total sleeping time (TST) less than 1 hour, possibly because of insomnia; and 48 patients whose ages less than 20 or more than 85 years old, data of 616 patients were used for further study. By further considering 76 patients with TST < 4 h, a group of 540 patients with TST ≥ 4 h was used to study the effect of insufficient sleeping time. Alice 4 PSG recorder (Respironics Inc., USA) was used to monitor patients with suspected OSA and to record their PSG data. After statistical analysis and feature selection, models built based on support vector machine (SVM) were then used to diagnose moderate and moderate to severe OSA patients with a threshold of AHI = 30 and AHI = 15, respectively.

**Results:**

The SVM models designed based on the oxyhemoglobin desaturation index (ODI) derived from oximetry measurements provided an accuracy of 90.42-90.55%, a sensitivity of 89.36-89.87%, a specificity of 91.08-93.05%, and an area under ROC curve (AUC) of 0.953-0.957 for the diagnosis of severe OSA patients; as well as achieved an accuracy of 87.33-87.77%, a sensitivity of 87.71-88.53%, a specificity of 86.38-86.56%, and an AUC of 0.921-0.924 for the diagnosis of moderate to severe OSA patients. The predictive outcome of ODI to diagnose severe OSA patients is better than to diagnose moderate to severe OSA patients.

**Conclusions:**

Overnight pulse oximetry provides satisfactory diagnostic performance in detecting severe OSA patients. Home-styled oximetry may be a tool for severe OSA diagnosis.

## Background

Breathing-related sleep disorder is a spectrum of diseases including snoring, upper airway resistance syndrome and sleep apnea [1]. Several studies have explored that sleep apnea is associated with obesity, daytime fatigue, high blood pressure, and increased risk of cardiovascular morbidity [2,3]. Obstructive sleep apnea (OSA) is prevalent in 4% of men and 2% of women [4]; among them, up to 93% of women and 82% of men have not been diagnosed [5], resulting in an increased risk of 2–7 folds in causing motor vehicle crashes [6] and causing several chronic diseases, such as metabolic syndrome [7]; neurocognitive deficits [8], vigilance alteration and attentional decline [9]; and erectile dysfunction [10].

Although polysomnography (PSG) is regarded as the gold standard, it is time-consuming, labor-intensive, and expensive [11]. According to a recent report, waiting time for accessing to diagnose and treat patients with suspected OSA is lengthy even in the developed counties around Europe, Australia, the United States, and Canada [12]; for example, it was estimated that the waiting time for non-urgent referrals for a sleep study ranges from 0 to 48 months in UK, 2 weeks to 2 months in Belgium, 4 to 68 weeks in Australia, a few weeks to more than a year in the US, and 8 to 30 months in Canada [12]. The waiting time ranges from 3 to 7 days in our hospital. Hence, other devices which are cheap, safe, and accurate; readily and easily accessed; and have no risk or side effects to the patients are needed for decreasing waiting time and cost for OSA diagnosis [11]. In addition to labor-intensive, time-consuming, and high examination cost, PSG also has other limitations, such as technical expertise is required and timely access is restricted [12]. Thus, home pulse oximetry has been proposed as a valuable screening tool, although its effectiveness in screening patients with OSA has been debated for several years [13]. A number of studies have assessed its usefulness, but sometimes with conflicting results [14-16]. For example, home overnight oximetry was found to be not very correlated with PSG for testing children [17] and to be considerable night-to-night variability for aged patients with chronic obstructive pulmonary disease (COPD) [18]. However, Brouillette and colleagues reported that oximetry could be used to diagnose OSA for children with a positive predictive value of 97% [19].

It was reported that total sleeping time (TST) and sleep efficiency were significantly smaller for the first night in-home PSG compared to the 2nd and 3rd nights [20]. Night-to-night variability of AHI greater than 5 among 21% of the study patients had also been observed [21]. Furthermore, Newell et al [22] showed that TST, sleep efficiency, and AHI were significantly different between 2 consecutive nights of PSG recording for 4 individual groups of subjects, including sleep-related breathing disorders, insomnia, movement and behavioral disorders, and healthy control, indicating that AHI might be affected by the TST.

Patients who do not have enough sleep time during the examination might have great influence on the diagnosis of OSA severity. Although Lam and colleagues excluded subjects with TST less than 4 hours [7], the reason why these data were removed was not reported. In this study, we compared two groups of subjects, one with TST less than 4 hours and another with TST more than 4 hours.

The support vector machine applied to oximetry for the diagnosis of OSA has been little explored. Recently, SVM was used to design classifiers for the diagnosis of OSA patients based on the data acquired from only 149 and 100 recruited subjects, respectively, by Al-Angari et al. [23] and Marcos et al. [24]. Marcos et al. [24] designed an SVM classifier using Gaussian kernel to classify spectral features of SaO_2_ signals. The subjects divided into 60 normal and 89 OSA patients without considering the severity were recruited in their study. Al-Angari et al. [23] adopted linear kernel and second-order polynomial kernel to design the SVM classifiers for discriminating 1-mimute epochs of OSA segments from the normal segments, and to discriminate patients from the normal subjects. The subjects were divided into 50 normal and 50 OSA patients. Only accuracy, sensitivity, and specificity were evaluated in both studies, while areas under ROC curve (AUC) were not calculated. In the present study, the PSG data of 616 patients with possible OSA were used to design SVM classifiers for the diagnoses of severe and moderate to severe OSA patients.

The objectives of this study are to (1) validate nocturnal oximetry for the screening of high-risk OSA patients using oxyhemoglobin desaturation index (ODI) to detect severe and moderate-to-severe patients; (2) consider the effect of patients with TST less than 4 hours compared with those having longer TST during PSG examination; and (3) determine the best kernel and features used for constructing the SVM classifiers to discriminate the OSA patients from the normal subjects.

## Methods

### Study subjects

A total of 699 patients with possible OSA were recruited and tested using PSG device for overnight recording at the Sleep Center of China Medical University Hospital after written informed consents from Jan. 2004 to Dec. 2005. By excluding 23 patients with total recording time less than 3 hours, 12 with poor oximetry recordings, poor EEG signals, or respiratory artifacts, and 48 subjects whose ages less than 20 or more than 85 years old (7), data of 616 patients were used for analysis. By further considering 76 patients with TST less than 4 hours [7], a group of 540 patients whose TST more than 4 hours was used to study the effect of insufficient sleeping time. The study has been approved by Institutional Review Board of China Medical University (Study No. DMR 96-IRB-17).

### PSG examination

Alice 4 PSG recorder (Respironics Inc., USA) was used to monitor and record patients with suspected OSA, during which a number of physiologic variables were measured and recorded during sleep. Physiologic sensors were used to record (1) EEG for detecting brain electrical activity and sleep staging on the basis of 30-sec epochs, (2) EOG and submental EMG for detecting eye and jaw muscle movement, (3) tibia EMG for monitoring leg muscle movement, (4) airflow (by using nasal cannula to measure oronasal pressur) for detecting breath interruption (1287 nasal flow, PTAF 2; Pro-Tech; Pittsburgh, PA), (5) inductance plethysmorgraphy for estimating respiratory effort (chest and abdominal excursion), (6) ECG for measuring heart rate, and (7) arterial oxygen saturation by using a finger probe oximeter (935 Oximeter Sensor; Respironics; Murrysville, PA).

PSG recordings were performed overnight (from 9:00 PM to 06:00 AM in general) in the sleep center for a minimum of 3 hours of light-on to light-off recording time when patients received OSA diagnoses. During the examination, the attending technicians on duty closely inspected the acquired signals of individual channels displayed on the monitor to check their signal quality. If the signal quality was poor or contained obvious artifacts, the technicians would check the instrument setup, cable connection, and electrode-skin contact on-site to ensure proper recording. The quality of PSG raw data was verified by an experienced physician of sleep medicine and scored by a technician certificated by the Taiwan Society of Sleep Medicine. During off-line analysis, the periods with artifacts or poor signal quality not meeting the diagnostic requirements, such as poor signals or artifacts in acquired oximetry, EEG, or respiratory data that did not allow the reading of the SpO2, sleep stages, or the respiratory events, were deleted from the recorded signals. For patients whose net total recording time less than 3 hours or TST less than 1 hour were arranged to receive another free examinations. Sleeping efficiency was calculated as TST divided by total recording time.

The oximeter was used to measure the SpO_2_ with a sampling rate of 1 Hz. Only the SpO_2_ signal acquired from pulse oximeter was used for ODI calculation without the knowledge of other PSG information. For pulse rates less than 112 beat/min, the SpO_2_ calculation is based on a four-beat exponential averaging. The averaging time is doubled to 8 beats for pulse rate greater than 112 beat/min, and is redoubled again for pulse rate more than 225 beat/min. Artifacts with changes of oxygen saturation between consecutive sampling intervals greater than 4%/s, and any oxygen saturation less than 50% were detected and eliminated by an automated algorithm [25].

An apnea was defined as a minimum of 10 seconds of airflow cessation, and a hypopnea was determined by a 30% reduction in airflow preceding a period of normal breathing for a minimum of 10 seconds and oxyhemoglobin desaturation (decrease in SpO_2_ ≧ 4%) [26]. Apnea-hypopnea index (AHI) was calculated as the total number of apneas (central, mixed, or obstructive) and hypopneas per hour of sleep. An arousal event was defined as an episode lasting 3 seconds or more with a return of α activity associated with a detectable increase in EMG activity [27]. Arousal index (AI) was the total number of arousals per hour of sleep. OSA patients were diagnosed as mild, moderate, and severe with an AHI greater than 5/h, 15/h, and 30/h, respectively.

The questionnaires including demographic information (age, gender, education, profession type, as well as smoking and drinking status), Epworth scaling score (ESS), as well as symptoms (snoring, drooling and teeth-grinding during sleep; feeling headache, tired, and dry mouth after wake-up; daytime drowsiness, memory problem, and traffic accidence caused by drowsiness when driving) and comorbidities (hypertension, diabetes, high blood lipid, high uric acid, stroke, heart attack, angina pectoris, asthma, thyroid disorder, and allergic rhinitis) related to OSA diagnosis were filled by the patients before PSG recording. Anthropometric parameters (weight, height, as well as waist, neck and hip circumferences) were measured and BMI calculated by the technicians.

### Oxyhemoglobin desaturation index derived from pulse oximetry measurement

Oxyhemoglobin desaturation index (ODI), represented as ODI*nb*, includes three components, i.e., threshold (*n*), baseline (*b*), and lasting time (fixed to *t =* 3 sec in this study) parameters. For threshold *n*, the amount of oxyhemoglobin desaturation *n*% decrease from the baseline was calculated. In this study, two different baselines, including the mean of all-night oxygen (*A*) [28] and the mean of the top 20% of oxyhemoglobin saturation values over the 1 min preceding the scanned oxyhemoglobin value (*T*) [16] were adopted. The lasting time parameter *t* = 3 was defined as that an oxyhemoglobin desaturation event with a minimum of 3 sec were continued. Finally, the ODI was calculated by dividing the total oxyhemoglobin desaturation counts with the total recording time (hours). For example, ODI4A calculated the amount of oxyhemoglobin desaturation events with a 4% decrease from the mean of all-night oxygen lasting for more than 3 seconds per hour. Because the oximetry is one of the PSG channels, we aim to investigate the accuracy, sensitivity, specificity, and area under ROC curve (AUC) of overnight oximetry for the diagnosis of moderate and moderate to severe OSA patients by ODI parameters, including ODI2 (ODI2T), ODI3 (ODI3T), ODI4T, and ODI4A, derived from oximetry measurements.

### Consideration of TST during PSG examination

Subjects who do not have enough sleep time during the examination might have great influence on the diagnosis of OSA severity. Although Lam and colleagues excluded subjects with TST less than 4 hours [7], the reason why these data were removed was not reported. In this study, we compared two groups of subjects, one with TST less than 4 hours and another one with TST more than 4 hours.

### Analytical techniques

Demographic, anthropometric, questionnaire, and PSG data of patients were analyzed using descriptive statistical analysis for calculating means and standard deviations of individual variables. Inferential statistical analyses including Pearson chi-square test and analysis of variance (ANOVA) were applied to detect significant variables for discriminating among normal subjects and mild, moderate, and severe OSA patients. Moreover, linear regression analysis and Bland-Altman plot were performed to compare correlations and agreements between ODI variables (ODI2, ODI3, ODI4T, and ODI4A) and AHI. Analysis of ROC curves were performed to set the cutoff values of different ODI parameters that best discriminate moderate and moderate/severe OSA patients from normal and mild subjects. Furthermore, we tested and compared various combinations of neck circumference (NC), body mass index (BMI), ESS, and ODI variables in the diagnosis of severe and moderate to severe patients based on 2 thresholds, AHI = 30 and AHI = 15, respectively. Finally, support vector machine (SVM), an artificial technique, was used to design computer-assisted diagnostic systems based on parameters derived from the oximetry measurements only by excluding signals acquired from other PSG channels. Comparisons of the diagnostic performance of traditional oximetry parameters with those from the SVM models were also conducted.

### Support vector machine

The SVM technique is a useful technique for data classification and regression, which has become an important tool for machine learning and data mining. In general, SVM has better performance when competed with existing methods, such as neural networks and decision trees [29-31]. Recently, application of SVM in medicine has grown rapidly. For examples, it has been applied in prediction of disulfide bonding patterns in proteins [32], discrimination of malignant and benign cervical lymph nodes [33], disease diagnosis using tongue images [34], diagnoses of cardiovascular disease [35] and breast cancer [36], and predictions of successful ventilation weaning [37]. A special property of SVM is that it can simultaneously minimize the empirical classification error and maximize the geometric margin. Its goal is to separate multiple clusters with a set of unique hyperplanes having greatest margin to the edge of each cluster to reduce misclassification induced by noise, where each hyperplane separating two clusters is not unique for ordinary linear classifiers. Recently, SVM was also used to design classifiers for the diagnosis of OSA patients [23,24].

## Results

### Demographic and clinical characteristics of participants

The tested subjects were divided into 4 groups based on the AHI for statistical analysis. Table 1 shows the demographic and clinical characteristics of participants and compares the statistic results among 4 patient groups. Regarding the influence of gender in OSA severity, the proportion of male patients increases significantly following the OSA severity (Chi-square test, *p* < 0.001). Demographic and anthropometric variables (age, BMI, and NC), ESS obtained from the questionnaire, and variables derived from oximetry (ODI and heart rate), reaching a level of significance (ANOVA, *p* < 0.01) among two or three stages, were selected as potential variables for designing the SVM classifiers to detect severe (30≦AHI) and moderate to severe (15≦AHI) patients. It was found that only ODI could be used to differentiate all 4 different groups. Variables, including TST, sleep latency, arousal count, and arousal index, derived from EEG and/or EMG, were also calculated and compared among 4 groups of participants. It can be observed that the TST is lower while the arousal count and arousal index are higher for more severe patients.

**Table 1:** **Statistic tests of demographic, questionnaire, and PSG data obtained from patients across four stages of severity based on AHI value**

**Severity** ^**a**^	**All**	**(1) Normal**	**(2) Mild**	**(3) Moderate**	**(4) Severe**	**Statistic test** ^**b**^
Patient No. (%)	616 (100%)	72 (11.7%)	127 (20.6%)	132 (21.4%)	285 (46.3%)	
Gender (%)^‡^						
Female	141 (22.9%)	32 (44.4%)	49 (38.6%)	31 (23.5%)	29 (10.2%)	*χ* ^2^ = 62.801, *p* < 0.001
Male	475 (77.1%)	40 (55.6%)	78 (61.4%)	101 (76.5%)	256 (89.8%)	
Age^***^	45.2 ± 12.7	36.2 ± 10.2	42.1 ± 11.5	45.9 ± 12.8	48.4 ± 12.5	(1) < (2),(3),(4); (2) < (4)
BMI^***^	26.74 ± 4.24	23.88 ± 3.15	25.60 ± 3.75	26.18 ± 3.68	28.22 ± 4.33	(1),(2),(3) < (4)
NC^***^	39.08 ± 3.65	36.24 ± 3.25	37.53 ± 3.52	38.69 ± 3.19	40.79 ± 3.10	(1) < (3); (1),(2),(3) < (4)
ESS^**^	9.21 ± 5.24	7.67 ± 5.16	8.54 ± 4.77	9.19 ± 5.25	9.93 ± 5.34	(1) < (4)
ODI parameter						
ODI2^***^	37.58 ± 23.50	10.25 ± 7.18	19.81 ± 10.57	29.60 ± 12.31	56.11 ± 18.94	(1) < (2) < (3) < (4)
ODI3^***^	26.16 ± 23.93	3.10 ± 2.78	8.18 ± 6.02	15.54 ± 8.87	44.92 ± 22.37	(1) < (2) < (3) < (4)
ODI4T^***^	20.44 ± 23.06	1.28 ± 1.43	4.18 ± 3.80	9.52 ± 7.01	37.58 ± 23.66	(1) < (2) < (3) < (4)
ODI4A^***^	24.65 ± 26.00	1.14 ± 1.41	5.20 ± 3.94	12.01 ± 7.62	45.10 ± 24.97	(1) < (2) < (3) < (4)
Heart Rate	73.32 ± 11.02	70. 31 ± 8.45	71.05 ± 10.61	72.64 ± 9.50	75.41 ± 11.97	(1),(2) < (4)
TST (min)^***^	309.0 ± 63.4	317.8 ± 56.3	331.3 ± 48.9	307.6 ± 58.5	297.5 ± 69.8	(3),(4) < (2)
Latency (min)^***^	19.6 ± 21. 1	27. 6 ± 33.0	16.2 ± 13.6	22.4 ± 23.2	17.9 ± 18.2	(2),(4) < (1)
Arousal count^***^	153.9 ± 99.1	95.3 ± 55.6	103.0 ± 52.3	121.1 ± 52.3	206.5 ± 112.4	(1),(2),(3) < (4)
Arousal Index^***^	33.6 ± 20.7	19.3 ± 10.8	20.6 ± 10.1	26.6 ± 11.2	46.3 ± 21.9	(1),(2),(3) < (4); (1) < (3)

^a^Normal: AHI < 5; Mild: 5≦AHI < 15; Moderate: 15≦AHI < 30; Severe: AHI≧30.

^b^ANOVA test with ^*^ *p* < 0.05, ^**^ *p* < 0.01, and ^***^ *p* < 0.001; Pearson Chi-square test with ^‡^ *p* < 0.001.

### Discrimination of normal, mild, moderate, and severe participants

Table 2 shows the confusion matrices of multiclass classifiers in the discrimination of 4 groups of participants using ODI parameters derived from the oximetry measurements for two datasets including 616 (dataset 1) and 540 (dataset 2) subject data, respectively. ODI parameters can provide a total predictive accuracy of 71.27% and 73.52% for dataset 1 and dataset 2, respectively. The sensitivities of SVM models in diagnosing severe patients are 88.07% and 87.76% in dataset 1 and dataset 2, respectively, while the sensitivities are less than 75% for other 3 categories. The predictive accuracy is obtained by tenfold cross validation.

**Table 2:** **Confusion matrices for the classification of 4 groups with ODI used as predictor for two datasets containing 616 and 540 samples, respectively**

	**N**	**Normal**	**Mild**	**Moderate**	**Severe**	**Sensitivity (%)**
Dataset 1						
Normal	72	53	10	1	0	73.61
Mild	127	25	80	20	2	62.99
Moderate	132	3	48	55	26	41.67
Severe	285	1	11	22	251	88.07
Total Validation	616	Overall accuracy: 71.27%				
Dataset 2						
Normal	68	51	17	0	0	75.00
Mild	121	21	85	14	1	70.25
Moderate	114	3	39	53	19	46.49
Severe	237	0	10	19	208	87.76
Total Validation	540	Overall accuracy: 73.52%				

### Diagnosis of severe and moderate/severe OSA patients

Table 3 displays various combinations of NC, BMI, ESS, and ODI for designing predictive models in the diagnosis of severe OSA patients, and the prediction rates and AUC achieve 89.81-90.58% and 0.946-0.958, respectively. Diagnostic models designed using ODI parameters derived from pulse oximetry alone achieved prediction performance similar to other multiclass predictors designed using more variables. Models designed with ODI parameters provided a sensitivity of 87.36-89.87%, a specificity of 91.08-93.05%, an accuracy of 90.42-90.55%, and an AUC of 0.953-0.957 for the diagnosis of severe OSA patients in both datasets.

**Table 3:** **Diagnosis of severe patients with AHI = 30 as the threshold using different combination of salient features**

**Data**	**Multiple variables**					**Single variable**			
	**Predictive index (%)**	**NC, BMI, ESS, ODI**	**ODI, ESS**	**ODI, BMI**	**ODI**	**ODI2**	**ODI3**	**ODI4T**	**ODI4A**
Dataset 1 (N = 616)	Accuracy	90.42	90.58	90.58	90.42	87.2	89.5	87.8	89.8
	Sensitivity	88.07	87.01	86.31	87.36	91.6	86.0	85.7	90.6
	Specificity	92.44	93.65	94.25	93.05	83.3	92.4	89.7	89.1
	AUC	0.958	0.956	0.954	0.957	0.938	0.942	0.912	0.913
	Cutoff	-	-	-	-	27.2	18.4	11.2	13.7
Dataset 2 (N = 540)	Accuracy	90.18	90.37	89.81	90.55	87.6	90.0	88.5	89.3
	Sensitivity	86.07	88.18	88.60	89.87	91.6	86.6	88.7	89.5
	Specificity	93.39	92.07	90.75	91.08	84.4	92.7	88.4	89.1
	AUC	0.952	0.946	0.950	0.953	0.945	0.946	0.913	0.913
	Cutoff	-	-	-	-	27.2	18.4	10.4	13.7

Note: ODI is the combination of ODI2 and ODI4A.

Table 4 compares the diagnostic performance of various diagnostic models designed with different combination of variables for the diagnosis of moderate to severe patients. The sensitivity, specificity, accuracy, and AUC achieve 87.71-89.97%, 83.08-86.56%, 86.85-88.14%, and 0.921-0.941, respectively. Again, ODI alone provides a similar predictive accuracy of 87.33-87.77%, a sensitivity of 87.71-88.53%, a specificity of 86.38-86.56%, and an AUC of 0.921-0.924, respectively, for the diagnosis of moderate to severe OSA patients.

**Table 4:** **Diagnosis of moderate to severe patients with AHI = 15 as the threshold using different combination of salient features**

**Data**	**Multiple variable**					**Single variable**			
	**Predictive index (%)**	**NC, BMI, ESS, ODI**	**ODI, ESS**	**ODI, BMI**	**ODI**	**ODI2**	**ODI3**	**ODI4T**	**ODI4A**
Dataset 1 (N = 616)	Accuracy	86.85	87.82	87.01	87.33	87.2	89.5	87.8	89.8
	Sensitivity	88.67	89.87	87.95	87.71	91.6	86.1	85.7	90.6
	Specificity	83.08	83.58	85.07	86.56	83.3	92.4	89.7	89.1
	AUC	0.938	0.935	0.927	0.921	0.918	0.920	0.870	0.869
	Cutoff	-	-	-	-	21.2	9.5	7.3	8.5
Dataset 2 (N = 540)	Accuracy	87.96	88.14	87.59	87.77	87.6	90.0	88.5	89.3
	Sensitivity	89.39	89.97	88.82	88.53	91.6	86.6	88.7	89.5
	Specificity	85.34	84.81	85.34	86.38	84.4	92.7	88.4	89.1
	AUC	0.941	0.939	0.940	0.924	0.925	0.927	0.872	0.875
	Cutoff	-	-	-	-	21.2	9.5	7.3	7.3

Note: ODI is the combination of ODI2 and ODI4A.

**Figure 1:**
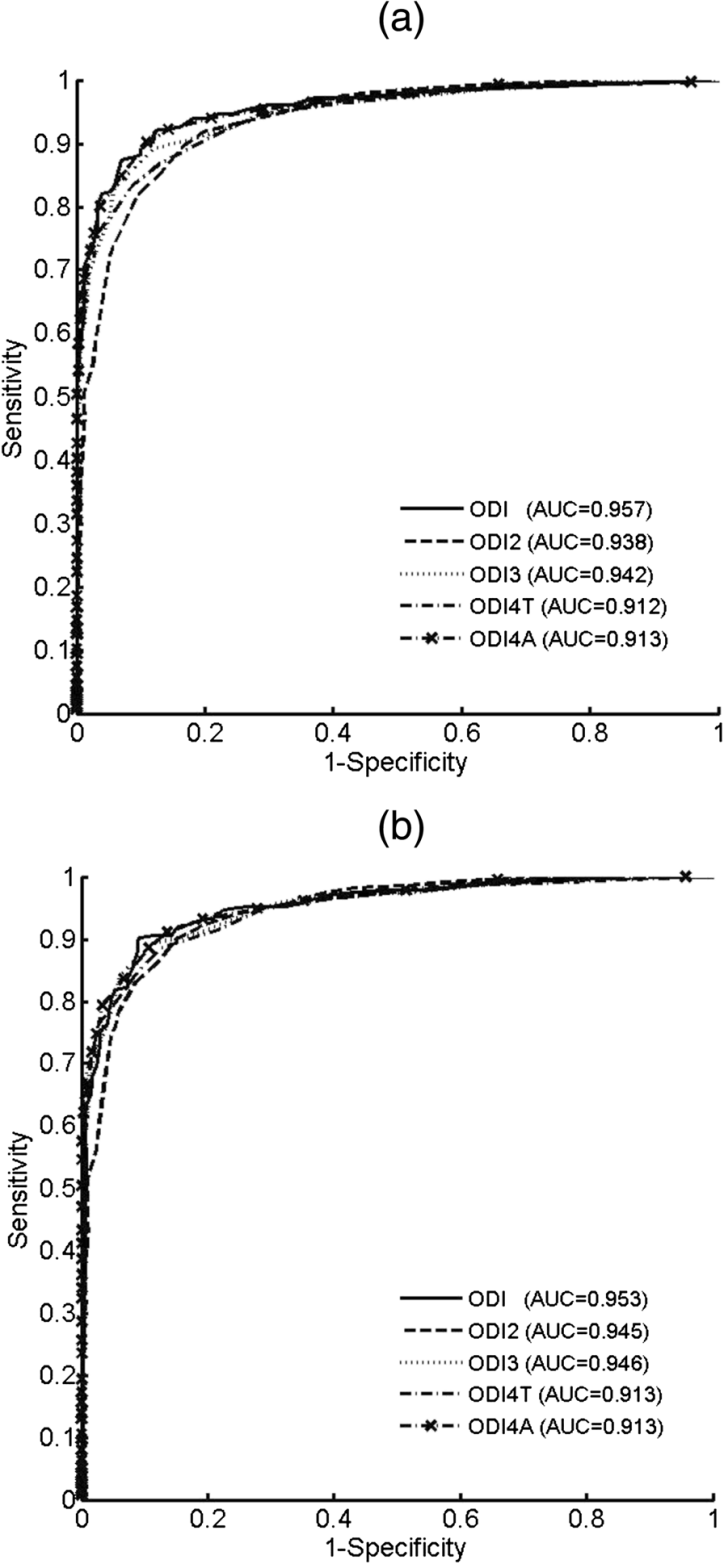
**ROC curves of ODI parameters for the diagnosis of severe and moderate/severe OSA patients with thresholds (a) AHI=30 and (b) AHI=15, respectively.**

Figure 1 illustrates and compares the ROC curves of models designed using traditional ODI variables (ODI2, ODI3, ODI4T, and ODI4A) and their combinations (ODI2 and ODI4A). In addition to higher accuracy (Table 3), the AUCs of the SVM models designed using 2 ODI variables are greater than other single-variable models, as shown in Figure 1(a) and 1(b), indicating its capability in diagnosing severe OSA patients outperforms other traditional models. Regarding the diagnosis of moderate to severe patients, as shown in Table 4, the multiple-variable SVM models and traditional single-variable models exhibit similar diagnostic performance.

### Cutoff values for diagnosis of OSA patients using ODI variables

The cutoff values of single-variable models for the diagnosis of severe and moderate to severe OSA patients are shown in Tables 3 and 4, respectively. The optimal cutoff values for severe patient diagnosis are 27.2, 18.4, 11.2, and 13.7, respectively, in dataset 1, as well as 27.2, 18.4, 10.4, and 13.7, respectively, in dataset 2 for ODI2, ODI3, ODI4T, and ODI4A. The cutoff values were decreased to 21.2, 9.5, 7.3, and 8.5 for dataset 1 as well as 21.2, 9.5, 7.3, and 7.3 for dataset 2 when making diagnosis of moderate to severe patients using ODI2, ODI3, ODI4T, and ODI4A, respectively.

### Effect of less TST

Table 5 shows that the number and percentage of subjects with different OSA severity, exhibiting significant difference between patients with TST less than 4 hours and those with TST more than 4 hours (*p* < 0.01). As indicated in the table, the percentage of severe patients in the group of TST < 4 h group (63.2%) is higher than the TST ≥ 4 h group (43.9%), while it is opposite by considering the cumulated normal and mild subjects (13.2% vs 35.0%). On the other hand, the percentages of moderate patients are very close between two groups (23.9% vs 21.1%). No significant difference (*p* > 0.05) was observed between two groups regarding anthropometric variables (BMI and NC), questionnaire (ESS), and HR, while significant difference was found for age (*p* < 0.001), ODI4A (*p* < 0.05), and AHI (*p* < 0.01). Interestingly, except ODI4A, other ODI parameters, i.e., ODI2, ODI3, and ODI4T, exhibited no significant difference (*p* > 0.05) between two different TST groups. It mimics ODI4A is more sensitive than ODI2, ODI3, and ODI4T in discriminating patients with less TST from those with more TST. Furthermore, as shown in Table 5, patients with less TST demonstrate significantly greater latency (*p* < 0.001) and arousal index (*p* < 0.05); however, the arousal counts is significantly smaller (*p* < 0.001). We suspected that data recorded from subjects who do not have enough sleeping time might not be reliable for analysis and should be excluded. An additional PSG examination should be conducted to draw more solid diagnostic conclusion.

**Table 5:** **Comparison of statistic results of demographic, anthropometric, questionnaire, and PSG variables between patients whose TST < 4 h and those with TST ≥ 4 h**

**Severity** ^**a**^	**All**	**TST < 4 h**	**TST ≥ 4 h**	**Statistic test (** ***p*** **)** ^**b**^
AHI^**^	34.07 ± 28.11	43.52 ± 26.81	32.74 ± 28.06	0.002
Severity^†^	616 (100%)	76 (12.34%)	540 (87.66%)	
Normal	72 (11.7%)	4 (5.3%)	68 (12.6%)	*χ* ^2^ = 15.4
Mild	127 (20.6%)	6 (7.9%)	121 (22.4%)	*p* < 0.01
Moderate	132 (21.4%)	18 (23.9%)	114 (21.1%)	
Severe	285 (46.3%)	48 (63.2%)	237 (43.9%)	
Age^**^	45.2 ± 12.7	52.20 ± 14.67	44.17 ± 12.16	0.001
BMI	26.74 ± 4.24	27.14 ± 4.37	26.68 ± 4.22	0.370
NC	39.08 ± 3.65	39.65 ± 3.56	39.06 ± 3.42	0.161
ESS	9.21 ± 5.24	9.43 ± 5.48	9.19 ± 5.22	0.599
ODI parameter				
ODI2	37.58 ± 23.50	41.48 ± 19.42	37.03 ± 23.98	0.073
ODI3	26.16 ± 23.93	27.53 ± 19.18	25.96 ± 24.53	0.521
ODI4T	20.44 ± 23.06	19.84 ± 17.99	20.53 ± 23.70	0.767
ODI4A^*^	24.65 ± 26.00	31.16 ± 24.23	23.73 ± 26.13	0.015
Heart Rate	73.32 ± 11.02	73.85 ± 12.49	73.24 ± 10.79	0.703
TST (min)^**^	309.0 ± 63.4	180.2 ± 53.3	327.1 ± 39.1	0.002
Latency (min)^***^	19.6 ± 21. 1	41.5 ± 40.2	16.6 ± 14.4	<0.001
Arousal count^***^	153.9 ± 99.09	103.2 ± 65.3	161.0 ± 101.0	<0.001
Arousal Index^*^	33.6 ± 20.7	39.3 ± 22.4	32.8 ± 20.3	0.011

^a^Normal: AHI < 5; Mild: 5≦AHI < 15; Moderate: 15≦AHI < 30; Severe: AHI≧30.

^b^ANOVA test with ^*^ *p* < 0.05, ^**^ *p* < 0.01, and ^***^ *p* < 0.001; Pearson Chi-square test with ^†^ *p* < 0.001.

## Discussion and conclusion

Prediction of OSA using questionnaires, demographics, clinical features, and physiological examination has been extensively studied in the last decade [11,38]. Goncalves and colleagues used Epworth sleeping scale (ESS), the sleeping disorders questionnaire, the Beck depression inventory, the medical outcome study 36-item short form health survey (SF-36), and a questionnaire on driving difficulties and accidents to evaluate subjects who were suspected to have breathing-related sleep disorders. Among them, ESS was found to be correlated to arousal index and apnea-hyponea index (AHI) [38], while contradicted by other investigators [11,39]. Khoo et al [40] used questionnaires, containing questions regarding snoring, choking, suffocating, and abrupt awaking during sleep, to study Asian populations, including Chinese, Malaysian, and Indian. It was found that the risk factors are similar to white populations in strong association of snoring and sleep apnea with male gender, older age, obesity, family history, and smoking [40]. In addition, demographic, clinical, and biochemical factors including age, sex, observed sleep apnea, fasting insulin, glycosylated hemoglobin A1_C_, and central obesity and lager NC were found to significantly increase the risk of higher AHI for the severely obese patients [39]. Strong correlation between patient self-perception and clinical examination, including Friedman tongue position grade and Friedman clinical staging, of OSA severity and AHI was also found [11]. In this study, ESS questionnaire was considered to be included for designing SVM classifiers for OSA diagnosis. As indicated in Table 1, it is only effective in discriminating the normal from the severe OSA patients. In contrast, all ODI parameters are able to discriminate the normal and different stages of OSA patients. As presented in Tables 3 and 4, diagnostic performance is not improved when ODI parameters are combined with ESS, nor is combined with BMI and/or NC. ODI parameters provided by overnight oximetry measurements may become good predictors in the diagnosis of OSA, while demographic and questionnaire variables are not very helpful to elevate the prediction rate [41].

### Selection of ODI parameters for designing SVM models

The selection of variables for designing predictive models for OSA diagnosis is based on ANOVA analysis, linear regression analysis, and Bland-Altman plot. Although all variables shown in Table 1 reached a level of significance in discriminating 4 groups of participants when tested with ANOVA, only NC, BMI, and ODI parameters were considered as potential predictors after post-hoc analysis in discriminating severe patients from the other groups. In contrast, all the ODI parameters are able to discriminate any 2 groups of participants. The variables derived from PSG channels other than oximetry, including TST, sleep latency, arousal count, and arousal index, were not included, because they cannot be derived from the home oximetry.

**Figure 2:**
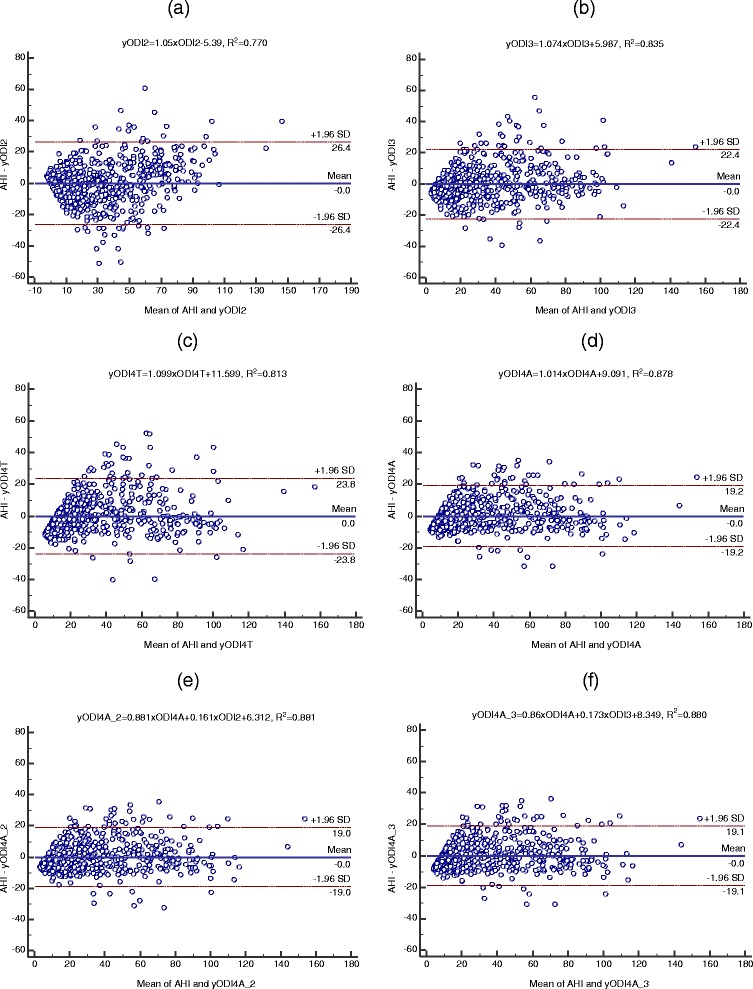
**Bland-Altman plots of AHI vs ODI parameters after linear regression analysis in dataset 1: (a) yODI2=1.05×ODI2-5.39, R** ^**2**^ **=0.770; (b) yODI3=1.074×ODI3+5.987, R** ^**2**^ **=0.835; (c) yODI4T=1.099×ODI4T+11.599, R** ^**2**^ **=0.813; (d) yODI4A=1.014×ODI4A+9.091, R** ^**2**^ **=0.878; (e) yODI4A_2=0.881×ODI4A+0.161×ODI2+6.312, R** ^**2**^ **=0.881; and (f) yODI4A_3=0.86×ODI4A+0.173×ODI3+8.349, R** ^**2**^ **=0.882.**

**Figure 3:**
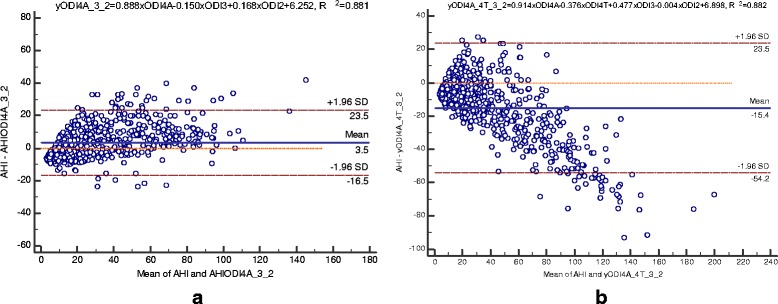
**Bland-Altman plots of AHI vs ODI parameters after linear regression analysis in dataset 1: (a) yODI4A_3_2=0.888×ODI4A-0.150×ODI3+0.168×ODI2+6.252, R** ^**2**^ **=0.881 and (b) yODI4A_4T_3_2=0.914×OD I4A-0.376×ODI4T+0.477×ODI3-0.004×ODI2+6.898, R** ^**2**^ **=0.882.**

Several ODI parameters, including ODI2, ODI3, ODI4T, and ODI4A, which were highly correlated with AHI were considered to be included for designing the SVM models. In addition to correlation, their agreements with AHI were also evaluated by Bland-Altman plots. After linear regression analysis, the obtained linear predictor functions were used to perform Bland-Altman plots to check their agreements with the gold standard AHI. As illustrated in Figure 2, Bland-Altman plots were compared among single and combined ODI parameters after linear regressions. The correlation coefficients (*R*^2^) of AHI versus ODI2, ODI3, ODI4T, and ODI4A were 0.770, 0.835, 0.813, and 0.878, respectively. The agreements of AHI with individual ODI parameters were evaluated by observing their means and differences. As exhibited in Figure 2 (a)-(d), the 95% limits of agreements (±1.96 SD) are ±26.4, ±22.4, ±23.8, and ±19.2, respectively, for ODI2, ODI3, ODI4T, and ODI4A with a mean of 0. ODI4A presented best agreement with AHI and was defined as the potential variable for combining with other variables to achieve better diagnostic performance. Figure 2 (e) and (f) demonstrate the Bland-Altman plots for ODI4A combined with ODI2 and ODI3, respectively. It can be found that the former combination (*R*^2^) = 0.881, ±1.96 SD = ±19.0) exhibits slightly better correlation and agreement with AHI than the latter (*R*^2^ = 0.880, ±1.96 SD = ±19.1) and ODI4A alone (*R*^2^ = 0.878, ±1.96 SD = ±19.2). The correlations and agreements were not improved by including more ODI parameters; for example, as shown in Figure 3, the correlation and agreements were slightly degraded for the combined ODI4A, ODI2, and ODI3 (*R*^2^ = 0.881 and ±1.96 SD = ±20.0) as well as for the combined ODI4A, ODI4T, ODI2, and ODI3 (*R*^2^ = 0.880 and ±1.96 SD = ±38.9) with significantly deviated means of 3.5 and −15.4, respectively. The analysis of ROC curves further validated that the combination of ODI4A and ODI2 achieved better diagnostic performance, especially for the diagnosis of severe OSA patients, than the predictors based on single ODI parameters.

### Comparison of multi-variable SVM classifiers and single-variable predictors

As shown in the confusion matrices (Table 2) resulted from multiclass SVM classifiers for the detection of normal, mild, moderate, and severe patients, the sensitivity of ODI variables (ODI 2 and ODI4A) achieves more than 87% in detecting severe OSA patients, while it is only 41-74% for the other three groups. The misclassification rates of severe and moderate patients classified as normal are 0.35% (1/285) and 2.27% (3/132), respectively, for dataset 1, as well as 0% and 2.63% (3/114), respectively, for dataset 2. The misclassified rates are only 1.38% and 0%, respectively, for normal subjects to be classified as moderate or severe when tested with dataset 1 and dataset 2. The results mimic that a system designed based on ODI parameters for diagnosing moderate or moderate/severe patients is expected to have great diagnostic performance.

The sensitivity in detecting severe patients is 87.36%, which indicates that 12.64% of the severe patients will be treated as normal, mild, or moderate (Table 3). Further PSG test will be expected to confirm the diagnoses of those mild and moderate patients. On the other hand, the percentage of normal, mild and moderate patients who were diagnosed as severe is 6.95% (1-specificity). According to the results of our dataset indicated in Table 2, none of the normal subject was diagnosed as severe, mimicking that the subjects being diagnosed as severe were mild or moderate OSA patients. It is acceptable for this misclassification (false-positive) since it was suggested that mild and moderate patients also need treatments using continuous positive airway pressure (CPAP) [42]. The oximetry can be effective to be used for diagnosing severe OSA patients. The predictive model is suitable to predict severe patients using cheaper and convenient oximetry, while the non-severe patients, including normal, mild, and moderate patients, need to be confirmed by more expensive PSG examinations. Cost-effectiveness analysis needs to be conducted to evaluate the strategy of home oximetry test followed by PSG examination for the diagnosis of OSA patients to reduce healthcare cost and medical facility usage.

Marcos et al. designed an SVM classifier using Gaussian kernel to classify spectral features of SaO_2_ signals [24]. The 149 subjects were divided into 60 normal and 89 OSA patients. The severity of OSA patients was not considered in their study. Since Gaussian kernel is deemed as an example of RBF kernel, the results were compared with our results obtained from RBF SVM classifier. The obtained accuracy, sensitivity, and specificity were 88.00%, 84.44%, and 93.33%, respectively, with an AUC of 0.921. In comparison, as presented in Tables 3, our results showed better performance with accuracy (≥90.42%), sensitivity (≥87.36%), specificity (≥91.08%), and AUC (≥0.953) in diagnosing severe OSA patients; and, as indicated in Table 4, similar performance with accuracy (≥87.33%), sensitivity (≥87.71%), specificity (≥86.38%), and AUC (≥0.921) in diagnosing moderate to severe patients.

Al-Angari et al. adopted linear kernel and second-order polynomial kernel to design the SVM classifiers for discriminating 1-mimute epochs of OSA segments from the normal segments, and to discriminate patients from the normal subjects [23]. Only 100 subjects (50 normal subjects and 50 OSA patients) were recruited in this study, lacking the representativeness of the study samples. The best accuracies achieved for the SVM classifiers designed based on the oxygen saturation features are 80.10% (Sensitivity: 60.9%; Specificity: 94.1%) and 95% (Sensitivity: 100%; Specificity: 90.2%) for epoch classification and subject classification, respectively. No AUC value was provided for both classifications.

Schlotthauer et al. [43] adopted a novel method based on the empirical mode decomposition to detect oxyhemoglobin desaturation events from the recorded pulse oximetry signals. ODI was then calculated from the oxyhemoglobin desaturation events to detect moderate OSA syndrome with a cutoff ODI value of 18.521, achieving a sensitivity, specificity, and AUC of 0.851, 0.853, and 0.923, respectively. Its diagnostic performance is slightly worse than the predictor ODI3 and the SVM model designed using combined ODI parameters, i.e., ODI2 and ODI4A, but is similar to the single-variable predictors, ODI2, ODI4T, and ODI4A (Table 4).

As shown in Table 6, diagnostic performance of SVM models embedding different kernels used for the diagnosis of severe OSA patients is compared. SVM models embedding RBF kernels present slightly better diagnostic performance than models designed with linear or polynomial kernels.

**Table 6:** **Comparison of diagnostic performance for SVM models with different kernels in the diagnosis of moderate and severe patients with AHI = 30 as the threshold using ODI features**

**Data**	**Predictive index (%)**	**Linear**	**Polynomial**	**RBF**
Dataset 1 (N = 616)	Accuracy	90.25	90.09	90.42
	Sensitivity	87.36	90.17	87.36
	Specificity	92.74	90.03	93.05
Dataset 2 (N = 540)	Accuracy	89.62	89.25	90.55
	Sensitivity	89.45	89.45	89.87
	Specificity	89.76	89.10	91.08

### Effect of less TST

By considering the TST, as depicted in Table 5, age, ODI, and AHI exhibit significant difference between patients with TST less than 4 hours and those with TST more than 4 hours. It indicates that older subjects and more severe OSA patients with higher ODI and AHI tend to have less sleeping time. There are two possible explanations of this finding: (1) some severe patients tend to sleep less because of frequent apnea or hypopnea occurrences and (2) data recorded from subjects who do not have enough sleeping time might not be reliable for further analysis. Regarding the first possibility, the frequent occurrences of apnea or hypopnea after having fallen asleep induces insomnia for severe patients. The environmental change might also be the reason for causing insomnia [44]. By observing the latency time in Table 5, significant difference (*p* < 0.001) can be found between two groups. Subjects with less sleeping time demonstrate greater latency. Although arousal count for TST less than 4 hours group is significantly smaller than those with TST more than 4 hours group (*p* < 0.001); however, the arousal index is significantly greater (*p* < 0.01). We suggest that subjects who were diagnosed as normal but didn’t take enough sleeping time in the sleeping center might be caused by environmental change and should have another PSG examination to confirm OSA diagnosis. By using oximeter to test OSA at home may eliminate such variation.

With regard to the second possibility, the data collected from the 76 (12.34%) subjects who had TST less than 4 hours were removed, resulting in a total of 540 subjects. To compare the accuracy in the discrimination of 4 different groups of subjects (Table 2), a greater increase in detecting mild (62.99% vs 70.25%) patients can be observed, while it is only small difference in accuracy regarding detection of normal (73.61% vs 75.00%), moderate (41.67% vs 46.49%), and severe (88.07% vs 87.76%) groups. Moreover, as shown in Tables 3 and 4, there is no significant difference in diagnostic performance when diagnosing severe patients or moderate and severe patients. The effect of less sleeping time needs to be further investigated.

### Study limitations

Unlike PSG and single-lead ECG, the limitation of oximetry measurement is that it is unable to score sleep quality [45]. Standard PSG scores sleep quality based on EEG signal analysis by grading the sleep quality into 4 stages of continuum of depth during non-REM sleep. Thomas and colleagues reported that sleep could be identified as wake/REM, cyclic alternating pattern (CAP), and non-CAP based on the Fourier analysis of R-R interval series and its associated ECG-derived respiration (EDR) signal [45]. The main advantage of using a combination of oximetry parameters as the predictor is its great sensitivity (>90%) in the diagnosis of severe OSA patients with cheaper price compared to PSG.

It was reported that study subjects selected based on whether they has been referred for the index test instead of clinical symptoms tended to have lower accuracy, whereas studies with nonconsecutive inclusion of subjects, retrospective selection of data, and discrimination between healthy subjects and severe patients had significantly higher accuracy [46]. Although our data were collected consecutively from Jan. 2004 to Dec. 2015 and the moderate and severe OSA patients were discriminated with a clear threshold of AHI = 15 and AHI = 30, respectively, expecting not to overestimate the diagnostic accuracy, several limitations must be considered when interpreting the findings. First, this is a tertiary referral hospital and the subjects were recruited from the outpatients with suspicious OSA. Among the 616 subjects studied, only 72 were verified as normal accounting to only 11.69% of the total subjects recruited, which was much less than the moderate (21.43%) and severe (46.27%) patients. Moreover, the study subjects selected based on the referred PSG examinations instead of clinical symptoms tended to underestimate the diagnostic accuracy [46]. Second, all events found in PSG were verified by the technicians working in the sleep center. The variation occurred among different technicians cannot be avoided and neglected since each one has his/her subjective judgment. Therefore, designing an objective computer-assisted system to eliminate the loads and subjective opinions of individual technicians is warranted. Third, the oximetry is part of the gold standard PSG examination, which reduces the quality of the study and can artificially increase the diagnostic accuracy using the parameters derived from the measurement of home-style oximetry for its liability to induce errors and to couple noise artifacts [47]. Finally, due to the design of this study limiting the applicability of the results at home, valid conclusions cannot be drawn about the accuracy of the oximetry to detect or exclude OSA patients outside the sleep laboratory.

## Conclusion

Overnight pulse oximetry provides satisfactory diagnostic performance in detecting severe OSA patients. Home-styled oximetry may provide a tool for the diagnosis of severe OSA patients. In addition to ODI parameters (ODI2 and ODI4A), other variables derived from the oximetry, such as heart rate variability (HRV) may also be considered to be used for designing computer-assisted diagnostic system to improve the diagnostic performance.
